# Five‐minute whole‐heart coronary MRA with sub‐millimeter isotropic resolution, 100% respiratory scan efficiency, and 3D‐PROST reconstruction

**DOI:** 10.1002/mrm.27354

**Published:** 2018-07-29

**Authors:** Aurélien Bustin, Giulia Ginami, Gastão Cruz, Teresa Correia, Tevfik F. Ismail, Imran Rashid, Radhouene Neji, René M. Botnar, Claudia Prieto

**Affiliations:** ^1^ School of Biomedical Engineering and Imaging Sciences, King's College London London United Kingdom; ^2^ MR Research Collaborations, Siemens Healthcare Limited Frimley United Kingdom; ^3^ Escuela de Ingeniería, Pontificia Universidad Católica de Chile Santiago Chile

**Keywords:** accelerated imaging, coronary MR angiography, isotropic sub‐millimeter resolution, patch reconstruction, respiratory motion, variable‐density undersampling

## Abstract

**Purpose:**

To enable whole‐heart 3D coronary magnetic resonance angiography (CMRA) with isotropic sub‐millimeter resolution in a clinically feasible scan time by combining respiratory motion correction with highly accelerated variable density sampling in concert with a novel 3D patch‐based undersampled reconstruction (3D‐PROST).

**Methods:**

An undersampled variable density spiral‐like Cartesian trajectory was combined with 2D image‐based navigators to achieve 100% respiratory efficiency and predictable scan time. 3D‐PROST reconstruction integrates structural information from 3D patch neighborhoods through sparse representation, thereby exploiting the redundancy of the 3D anatomy of the coronary arteries in an efficient low‐rank formulation. The proposed framework was evaluated in a static resolution phantom and in 10 healthy subjects with isotropic resolutions of 1.2 mm^3^ and 0.9 mm^3^ and undersampling factors of ×5 and ×9. 3D‐PROST was compared against fully sampled (1.2 mm^3^ only), conventional parallel imaging, and compressed sensing reconstructions.

**Results:**

Phantom and in vivo (1.2 mm^3^) reconstructions were in excellent agreement with the reference fully sampled image. In vivo average acquisition times (min:s) were 7:57 ± 1:18 (×5) and 4:35 ± 0:44 (×9) for 0.9 mm^3^ resolution. Sub‐millimeter 3D‐PROST resulted in excellent depiction of the left and right coronary arteries including small branch vessels, leading to further improvements in vessel sharpness and visible vessel length in comparison with conventional reconstruction techniques. Image quality rated by 2 experts demonstrated that 3D‐PROST provides good image quality and is robust even at high acceleration factors.

**Conclusion:**

The proposed approach enables free‐breathing whole‐heart 3D CMRA with isotropic sub‐millimeter resolution in <5 min and achieves improved coronary artery visualization in a short and predictable scan time.

## 1 INTRODUCTION

Three‐dimensional (3D) whole‐heart coronary magnetic resonance angiography (CMRA) has shown significant potential for both diagnosis and characterization of coronary artery disease (CAD) without radiation exposure or the need for intravenous contrast.^1^, ^2^, ^3^ Previous studies have shown good diagnostic accuracy of conventional CMRA for the identification of significant CAD (defined as luminal stenosis >50%) in the proximal‐mid coronary segments compared to the non‐invasive gold‐standard computed tomography coronary angiography,^4^ demonstrating its effectiveness as a screening tool. However, the low spatial resolution of conventional CMRA and its anisotropy impedes the quantification of luminal stenosis and hinders visualization of distal segments.

To address this challenge, isotropic sub‐millimeter 3D CMRA is required for more accurate assessment of lesion severity and effective risk stratification of patients. However, such imaging is not clinically feasible with conventional fully sampled free‐breathing diaphragmatic‐navigated (dNAV) CMRA, used to minimize respiratory motion, caused by excessively long and unpredictable scan times because only a fraction of the acquired data is accepted for reconstruction (referred to as scan efficiency).^5^, ^6^ Several approaches have been proposed to compensate for respiratory motion and achieve 100% respiratory scan efficiency. 1D self‐navigation techniques^7^, ^8^, ^9^, ^10^, ^11^ repeatedly acquire the k‐space center to infer the translational superior–inferior respiratory‐induced motion of the heart. To reduce motion estimation errors caused by contribution from static tissues (e.g., chest wall), 2D and 3D image‐based navigators (iNAV) approaches have been recently proposed.^12^, ^13^, ^14^, ^15^ These methods use low‐resolution images to estimate and correct for 2D or 3D respiratory motion of the heart. Most of these approaches enable ∼100% scan efficiency resulting in ∼50% reduced scan time. In spite of these developments, scan times for sub‐millimeter resolution images remain lengthy.^16^ For example, at an average heart rate of 70 beats/min, a fully sampled whole‐heart 3D Cartesian CMRA scan at 0.9 mm^3^ isotropic resolution may take as long as 1 h using dNAV (5‐mm gating window) and assuming 50% scan efficiency, which may be reduced to ∼30 min using motion correction techniques with 100% scan efficiency.

Another approach to overcome the prohibitively long acquisition times in isotropic whole‐heart CMRA is to use undersampling techniques, such as parallel imaging (PI)^17^, ^18^, ^19^ and compressed sensing (CS).^20^, ^21^, ^22^ Sparsity of MR images has been extensively exploited with CS, which states that a measured signal can be accurately recovered from few samples, under the assumption that the measured signals are randomly sampled, the signal is sparse in some basis and a non‐linear reconstruction is used to enforce sparsity and data consistency.^23^ PI and CS have been combined to further reduce isotropic 3D whole‐heart CMRA^20^, ^24^ scan times. Recently, patch‐based image reconstructions exploiting local redundancies and low‐rank matrix structures have been introduced for MR reconstruction to lead to sparser representations.^24^, ^25^ By modeling the similarity of image patches through block‐matching, low‐rank representation and 3D filtering, 2D patch‐based reconstructions used in concert with dNAV acquisitions have been shown to outperform conventional CS CMRA by recovering better image details and edges, as well as exhibiting improved overall image quality.^24^, ^26^ However, these techniques have not been combined with respiratory motion correction and may suffer from residual aliasing artifacts for high acceleration factors, which may compromise the diagnostic value of the reconstructed coronary artery images.

In this study, we sought to achieve sub‐millimeter isotropic 3D whole‐heart Cartesian CMRA in a short and predictable scan time by combining 2D iNAV respiratory motion correction with variable density spiral‐like sampling and a novel 3D patch‐based undersampled reconstruction. The proposed 3D patch‐based reconstruction (3D‐PROST) further exploits the inherent redundancies of the complex 3D anatomy of the coronary arteries using an effective and efficient low‐rank framework.

## 2 METHODS

### 2.1 Whole‐heart image navigated undersampled 3D CMRA sequence

A prototype free‐breathing 3D whole‐heart, electrocardiogram (ECG)‐triggered, balanced steady‐state free precession (bSSFP) sequence with variable density Cartesian undersampling was implemented by extending a previously proposed spiral‐like Cartesian acquisition^27^ following a similar approach to Cheng et al.^28^ The k_y_‐k_z_ phase‐encoding plane is sampled following approximate spiral interleaves on the Cartesian grid with variable density along each spiral arm. One spiral arm is acquired per cardiac cycle and is then rotated from 1 cardiac cycle to the next one. The k_y_‐k_z_ plane is then segmented in 2 sets of concentric rings, the first defining the fully sampled k‐space center and the second representing the accelerated spiral branches. The phase‐encoding lines within each ring are sorted according to a defined increment angle from 0 ° to 360 ° as in Prieto et al.^27^ The sampling of the branches is accelerated exponentially from the k‐space center to its periphery. The size of the fully sampled k‐space center was optimized experimentally on several data sets (not reported here) and was set to 20% the size of k_y_ and k_z_ encoding directions. This undersampled trajectory ensures a pseudo‐random pattern through the cardiac cycle, resulting in incoherent aliasing that spreads irregularly in a noise‐like fashion.

**Figure 1:**
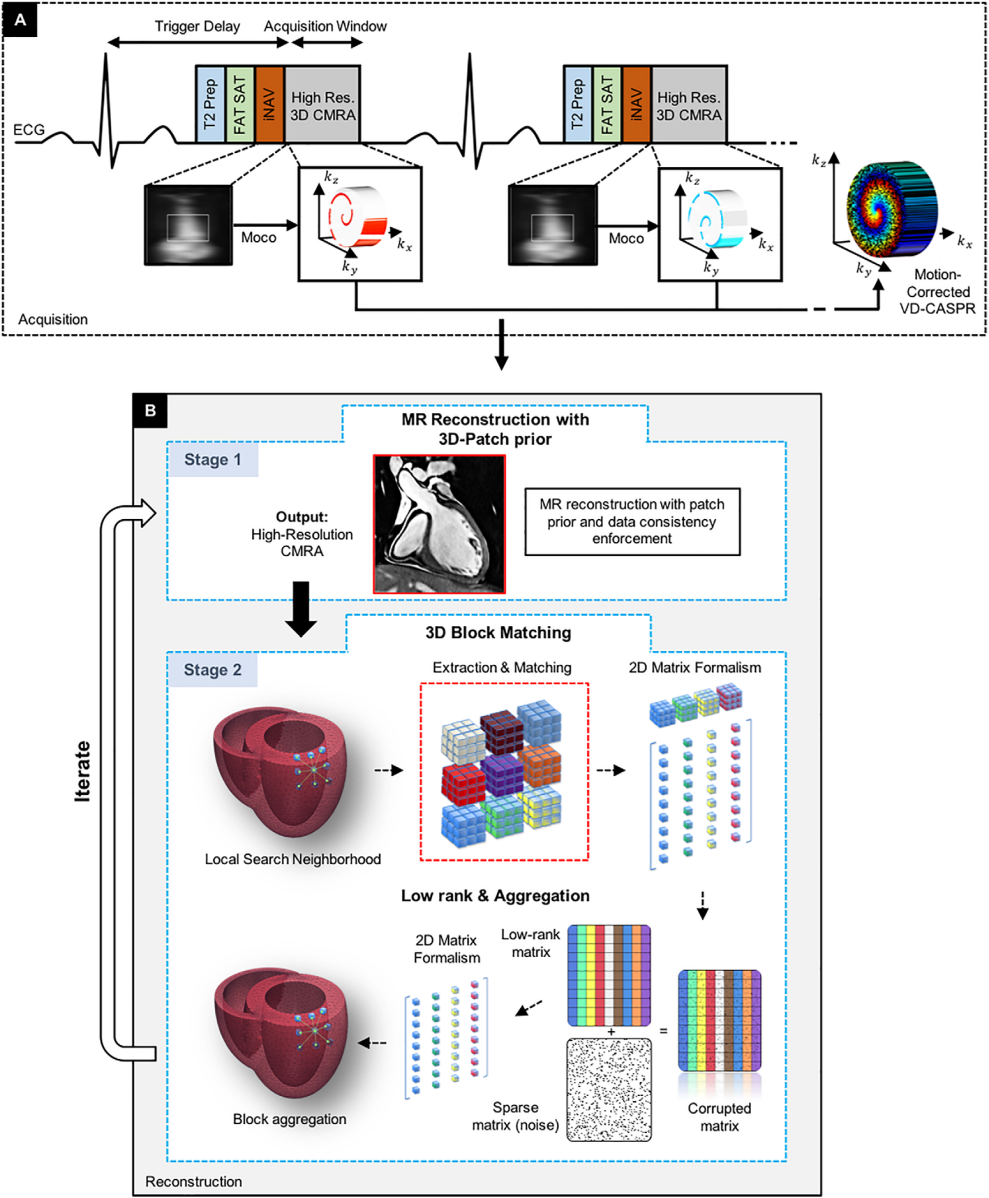
Acquisition and motion correction (A) is performed with an undersampled 3D variable density spiral‐like Cartesian trajectory (VD‐CASPR), preceded by 2D image navigators (iNAV), T_2_ preparation, and fat saturation pulses. iNAVs are used to estimate and correct for the beat‐to‐beat 2D translational respiratory‐induced motion of the heart (Moco). (B) 3D‐PROST reconstruction involves 2 stages of an augmented Lagrangian optimization scheme. In stage 1, image reconstruction is performed with patch prior and data consistency enforcement. In stage 2, image denoising is performed using 3D block‐matching, which groups similar 3D patches in the image, followed by a low‐rank approximation of each group using SVD shrinkage. The denoised volume from stage 2 is used in the reconstruction process in stage 1 as prior knowledge to regularize the reconstruction problem and further reduce noise

The acquisition of each spiral arm was preceded by a 2D iNAV to enable beat‐to‐beat 2D translational respiratory motion estimation/compensation (SI, superior‐inferior; RL, right‐left) and 100% scan efficiency. iNAVs were obtained by spatially encoding 14 startup echoes of the bSSFP CMRA sequence.^29^ 2D translational motion was estimated using a template‐matching algorithm,^30^ with the template manually selected around the heart during acquisition planning. Motion compensation was performed by modulating the k‐space data with a linear phase shift^31^, ^32^ to a reference position at end‐expiration. Motion estimation/compensation was performed before 3D‐PROST reconstruction and was implemented inline in the scanner software (Siemens Syngo MR, E11A, Siemens Healthcare, Erlangen, Germany). The proposed acquisition framework is depicted in the sequence diagram of Figure 1A.

### 2.2 3D patch‐based low‐rank RecOnSTruction (3D‐PROST)

The general formulation of sparse representation in terms of a redundant dictionary considers a 3D image m as the approximation m≈Dα, where D is a fixed dictionary and α a sparse vector satisfying the sparsity‐inducing condition ${\left\|\|\alpha \|\right\|}_{0}\leq T$, where the $l_{0}$‐norm counts the number of nonzero elements in α and T is a predefined threshold. Strictly speaking, the image m can be represented with a minimum number of sparse coefficients α in the redundant dictionary D. In this study, we propose a novel reconstruction algorithm that iteratively exploits the structure redundancy in the acquired data m to construct a specific dictionary D for each group of similar 3D patches.

The proposed 3D‐PROST scheme for isotropic CMRA reconstruction using 3D patch redundancy is formulated as the following unconstrained optimization on the sparse coefficients α 33:

(1) $${\text{argmin}}_{\alpha }\frac{1}{2}{\left\|\|AFS_{c}D\alpha -K\|\right\|}_{2}^{2}+\lambda {\left\|\|\alpha \|\right\|}_{0},$$

where $S_{c}$ are the known coil sensitivities for channel c, F is the Fourier transform, A is the sampling operator, K is the acquired multi‐channel k‐space data, and λ is the regularization parameter. By introducing a variable m and considering the acquisition model $E=AFS_{c}$, we transform Equation (1) into its equivalent constrained minimization problem

(2) $${\text{argmin}}_{m,\alpha }\frac{1}{2}{\left\|\|Em-K\|\right\|}_{2}^{2}+\lambda {\left\|\|\alpha \|\right\|}_{0}\;s.\;t.\;m=D\alpha ,$$

where m denotes the image to recover. One strategy to solve this minimization problem is to approximate Equation (2) using its augmented Lagrangian formulation,^34^ which implies minimizing the augmented Lagrangian L, defined below, with regards to m, α, and *b*

(3) $$\begin{array}{c} L_{3DPROST}\left(m,\alpha ,b\right)=\frac{1}{2}{\left\|\|Em-K\|\right\|}_{2}^{2}\; \end{array}+\lambda {\left\|\|\alpha \|\right\|}_{0}+\frac{\mu }{2}{\left\|\|m-D\alpha -b\|\right\|}_{2}^{2},$$

where b represents the Lagrange multiplier associated with the constraint “ m=Dα,” and μ≥0 is the penalty parameter. We solve Equation (3) using a variable splitting approach by alternating the minimization with respect to the image m (Optimization 1) and the sparse coefficients α (Optimization 2), followed by an update of the augmented multiplier b, and repeating these 3 steps until a convergence criterion is satisfied. The rationale behind this splitting approach is that each sub‐problem is now much simpler to solve than the original unconstrained problem in Equation (1).

#### 2.2.1 Optimization 1: MR reconstruction update

The first sub‐problem with regards to the variable m is a conventional MR reconstruction that incorporates the denoised volume Dα (obtained at the end of stage 2) as prior information, using l_2_‐norm regularization:

(4) $$\begin{array}{c} L_{MRecon}\left(m\right)={\text{argmin}}_{m}\frac{1}{2}{\left\|\|Em-K\|\right\|}_{2}^{2}\; \end{array}+\frac{\mu }{2}{\left\|\|m-D\alpha -b\|\right\|}_{2}^{2}.$$

Differentiating with respect to m, the residual gradient step is defined by:

(5) $$r=E^{H}Em-E^{H}K+\mu \left(m-\omega -b\right),$$

where the operator $E^{H}$ denotes the Hermitian transpose of E and ω=Dα represents the truncated singular value decomposition (SVD) reconstruction obtained at stage 2. For the initialization, ω and b were set to 0, which reduce the reconstruction problem to a standard iterative SENSE with Tikhonov regularization. We use the gradient descent optimization method to iteratively update the reconstructed volume m:

(6) $$m^{\left(t+1\right)}\leftarrow m^{\left(t\right)}-\beta r^{\left(t\right)},$$

where the relaxation parameter β can be updated iteratively or be set to a specific value (e.g., β=0.1) to ensure convergence.

#### 2.2.2 Optimization 2: 3D patch‐based denoising update

The second sub‐problem minimizes with respect to the sparse coefficients α and is given by

(7) $$\begin{array}{c} L_{Patch}\left(\alpha \right):\underset{\alpha }{\text{argmin}}\frac{1}{2} \end{array}{\left\|\|m-D\alpha -b\|\right\|}_{2}^{2}+\lambda {\left\|\|\alpha \|\right\|}_{0}.$$

Considering that sparse image coding is a local model representation and that neighboring patches in CMRA images are highly redundant, this optimization can be performed on an image patch basis approximating the l_0_‐norm by hard‐thresholding. A 3D patch $m_{k}$ in the previously reconstructed volume m of size N voxels is defined as a small 3D block of size $n^{3}$ voxels around the voxel at index k. We define the operator $R_{k}$ that extracts the patch $m_{k}$ from the image m: $m_{k}=R_{k}\left(m\right)$. Inversely, the image m can be recovered from its set of patches using:

(8) $$m=\left(\overset{N}{\underset{k=1}{\sum }}R_{k}^{H}\left(m_{k}\right)\right)./\left(\overset{N}{\underset{k=1}{\sum }}R_{k}^{H}R_{k}\right)=\left(\overset{N}{\underset{k=1}{\sum }}R_{k}^{H}\left(D_{k}\alpha_{k}\right)\right)./\left(\overset{N}{\underset{k=1}{\sum }}R_{k}^{H}R_{k}\right),$$

where the operator $R_{k}^{H}R_{k}$ is a matrix of same size as $m_{k}$ with all elements being 1 (i.e., averaging matrix) and the operator ./ denotes the element‐wise division. Equation (7) can therefore be rewritten as the following patch‐based minimization:

(9) $$\begin{array}{c} {\text{argmin}}_{\alpha_{k}}\frac{1}{2} \end{array}{\left\|\|m_{k}-D_{k}\alpha_{k}-b_{k}\|\right\|}_{2}^{2}+\lambda {\left\|\|\alpha_{k}\|\right\|}_{0}\;\;\;for\;k=1,\ldots ,N.$$

The key of an efficient resolution of this optimization problem lies in the good choice of the dictionary $D_{k}$ that induces the highest sparsity in its associated group of similar patches, and how accurately the sparse coefficients $\alpha_{k}$ can be recovered from this dictionary. In 3D patch‐based representation, we consider the self‐similarity as a 4D set of similar 3D patches $\left[m_{1},\ldots ,m_{L}\right]$, and its associated sparse coefficients $\left[\alpha_{1},\ldots ,\alpha_{L}\right]$ obtained from a specific dictionary. In previous studies, transform‐domain based on 3D fast Fourier transform were successfully used to promote sparsity in the self‐similarity group built from 2D patches, followed by hard‐thresholding and Wiener filtering to reduce the apparent blurring artifacts.^24^ To account for 3D patches, we reduce the complexity of the problem by concatenating each similar vectorized 3D patch into a 2D matrix. This 2D matrix, containing a high degree of similarity, exhibits a low‐rank structure that can be sparsely approximated using SVD. The low‐complexity self‐adaptive dictionaries *D*_*k*_ are therefore designed using SVD to sparsely represent each group of similar patches, where the sparse coefficients $\left[\alpha_{1},\ldots ,\alpha_{L}\right]$ are represented, for each group, by a few dominant singular values. Using the unitary property of the SVD, the minimization problem in Equation (9) is equivalent to minimizing with regards to the sparse coefficients the following equation^33^

(10) $$L_{Patch}^{k}(\alpha_{k}):\begin{array}{c} \underset{\alpha_{k}}{\text{argmin}}\frac{1}{2} \end{array}{\left\|\|{\tilde{\alpha }}_{k}-\alpha_{k}\|\right\|}_{2}^{2}+\lambda {\left\|\|\alpha_{k}\|\right\|}_{0},$$

where ${\tilde{\alpha }}_{k}$ are the sparse coefficients associated with $m_{k}-b_{k}={\tilde{m}}_{k}$ and λ>0 controls the strength of sparsity. The lower the parameter λ, the more accurate the reconstructed solution, at the price of reducing the sparseness. The solution of Equation (10) can be obtained using hard‐thresholding^35^

(11) $$\alpha_{k}^{*}=H_{\sqrt{2\lambda }}\left({\tilde{\alpha }}_{k}\right),$$

where $H_{\theta }\left(.\right)$ is the element‐wise hard‐thresholding operator, defined for a scalar v as: $H_{\theta }\left(v\right)=\theta .1_{\left|v\right|>\theta }$. In other words, any singular value $\begin{array}{c} {\tilde{\alpha }}_{k} \end{array}$ below $\sqrt{2\lambda }$ is set to 0. Note that only the singular values are modified, but the singular vectors are unperturbed. This step is repeated for each voxel in the volume m and the final denoised 3D volume ω=Dα is obtained by aggregating the multiple estimates $\begin{array}{c} D_{k} \end{array}\alpha_{k}^{*}$ at each voxel location k=1,…, N. The Lagrange multiplier is then updated at iteration t+1 as

(12) $$b^{\left(t+1\right)}\leftarrow b^{\left(t\right)}+\tau \left(\omega^{\left(t+1\right)}-m^{\left(t+1\right)}\right),$$

where the penalty parameter τ was set to 0.1 in all the experiments.

Optimizations 4 and 10 are processed iteratively to improve the accuracy of the reconstructed volume. A flowchart illustrating all steps is shown in Figure 1B. The effect of exploiting 3D redundancy, instead of focusing on 2D patches (2D‐PROST), is shown in Supporting Information Figure S1.

### 2.3 Image reconstruction implementation

Like most MR reconstruction algorithms, the performance of the 3D‐PROST technique relies on several parameters that need to be carefully tuned to get the best reconstruction. The central parameters of interest are the size of patch n, size of neighborhood window d, number of selected patches L, regularization parameters λ and μ, as well as the number of outer iterations (i.e., MR reconstruction and 3D denoising steps). The size of patch n controls the degree of structural information within each patch. On one hand, a large value of n would capture the most geometric information and leads to a higher level of denoising, whereas a small value would act as a local filter and would potentially reduce the denoising performance of the algorithm. The computational cost of the algorithm is highly dependent on this parameter as well. For all the experiments, we set the size of patches to be 5 × 5 × 5 voxels and the search window d to 14, which gives a good tradeoff between reconstruction quality and computation time. The number of selected similar patches L did not seem to affect the quality of the reconstructions, therefore we empirically set the value of L to 40, to avoid high computation cost and excessive memory requirements associated with large values of L. The performance of 3D‐PROST was evaluated by comparing reconstructions (not reported here) with several similarity thresholds λ and regularization parameter values μ. The optimal value ranges were determined, and we empirically set the values λ=0.1 and μ=0.3 in all experiments. Coil sensitivity maps were estimated from the fully sampled k‐space center using the adaptive coil combination technique^36^ and 4 outer iterations were chosen for all experiments as a good tradeoff between computational speed and reconstruction quality.

3D‐PROST reconstruction was performed offline on a workstation with a 16‐core Dual Intel Xeon Processor (2.3 GHz, 256 GB RAM) with the MR reconstruction step implemented in MATLAB (v7.1, The MathWorks, Natick, MA) and the 3D denoising step in C to reduce the computational time. The proposed 3D‐PROST reconstruction was compared to iterative SENSE (itSENSE)^37^ and a CS reconstruction with l_1_‐wavelet regularization, as implemented in the BART toolbox.^38^ The regularization parameter was carefully tuned and set to $\lambda_{CS}=0.01$ in all studies. The CS and itSENSE algorithms were stopped after 30 and 5 iterations, respectively, because preliminary testing revealed that these numbers of iterations led to the best reconstructions.

The feasibility of the proposed framework was tested in phantom at isotropic resolution 0.9 mm^3^ (see Supporting Information Figure S1) and in vivo experiments at 2 different isotropic spatial resolutions: 1.2 mm^3^ and 0.9 mm^3^. All experiments were performed on a 1.5T scanner (Siemens Magnetom Aera, Erlangen, Germany) using 18‐channel body and 32‐channel spine coils. Written consent was obtained from all participants before undergoing CMRA scans and the study was approved by the Institutional Review Board.

### 2.4 In vivo study

Ten healthy subjects (5 men and 5 women, mean age: 31 ± 8 y, range: 25–52 y) underwent ECG‐triggered free‐breathing whole‐heart CMRA using the proposed acquisition approach. Relevant scan parameters included: 3D bSSFP sequence, FOV = 320 × 320 × 86–115 mm^3^, FA = 90 °, T_2_‐preparation duration = 40 ms, subject‐dependent mid‐diastolic trigger delay and acquisition window (range, 90–130 ms). To ensure adequate fat suppression, a SPIR fat saturation pulse was applied before imaging with a constant flip angle of 130 °. A 2D iNAV preceded each spiral acquisition to achieve 100% scan efficiency and predictable scan time. 3D CMRA acquisitions were performed in the coronal plane.

#### 2.4.1 Impact of undersampling on reconstruction

The proposed 3D‐PROST reconstruction was evaluated for undersampling factors of ×5 and ×9 in acquisitions with isotropic 1.2 mm^3^ resolution in comparison to a fully sampled reference scan, which can be performed within a reasonable acquisition time at this resolution. Specific acquisition parameters for this study included: TE = 1.5 ms, TR = 3.4 ms, bandwidth per pixel = 875 Hz.

Acquired translational motion‐corrected data was reconstructed with itSENSE, CS, and the proposed 3D‐PROST. Reconstructed images were reformatted along the right (RCA) and left anterior descendent (LAD) coronary arteries and visible vessel length and vessel sharpness (first 4 cm and full length) were measured using Soap‐Bubble.^39^

Two experienced cardiologists (T.F.I. and I.R., 9 and 3 y of experience, respectively, SCMR level III certification), who were blinded to the reconstruction techniques, evaluated the quality of the reconstructed images. For each undersampling factor and each subject, the experts viewed the reformatted images (fully sampled, itSENSE, CS, and 3D‐PROST) in a random order and ranked the quality of both RCA and LAD from worst (score 1) to best (score 4). In addition, reconstruction quality based on RCA and LAD delineations was assessed using a 4‐point scoring system with 1 indicating uninterpretable CMRA images; 2 indicating poor image quality (blurred edges, noise and residual artifacts, low confidence in the diagnosis); 3 indicating acceptable image quality (RCA/LAD adequately visualized, only mildly blurred edges); and 4 indicating fully diagnostic images (excellent image quality with sharply defined coronary borders).

Vessel sharpness and length were assessed separately for the RCA and LAD using a two‐tailed Student's t‐test. Statistical significance of the expert quality scores was evaluated using a Wilcoxon signed rank test. *P*‐values of 0.016 or less were considered to be statistically significant after Bonferroni correction for multiple comparisons. The fully sampled CMRA acquisition served as the reference image for both the qualitative and quantitative analyses.

#### 2.4.2 Impact of resolution on coronary visualization

A second set of in vivo experiments was carried out to investigate the performance of the proposed approach for sub‐millimeter isotropic 3D CMRA acquisition. For this purpose, the 10 healthy subjects were also scanned at a 0.9 mm^3^ isotropic resolution with undersampling factors of 5 and 9 using the proposed approach. The fully sampled acquisition was prohibitively long for this resolution (∼40 min). Imaging parameters for this study were set to the same values as for the previous in vivo experiment except for TE/TR = 1.6/3.7ms and bandwidth per pixel = 890 Hz. The impact of resolution on coronary artery visualization was assessed by comparing the images acquired with 0.9 mm^3^ isotropic resolution with the fully sampled 1.2 mm^3^ isotropic resolution acquisition and its corresponding 5‐fold and 9‐fold undersampled reconstructions.

For each 3D‐PROST reconstructed image and both undersampling factors, visualization of proximal, middle, and distal segments of the RCA and LAD were identified and scored on a 3‐point scoring system for coronary visualization by the 2 experienced cardiologists. The coronary artery segments were graded as follows: 0, not visible; 1, partial visibility; and 2, excellent visualization of the coronary artery segment. To test for statistical differences, a Wilcoxon signed rank test was used. The visible vessel length was also measured for both the RCA and LAD and tested for statistical differences with a two‐tailed Student's t‐test. Statistically significant differences were defined as *P* < 0.025 after Bonferroni correction for multiple comparisons.

## 3 RESULTS

Free‐breathing whole‐heart CMRA acquisitions and reconstructions were completed successfully in all subjects. The mean heart rate was 60 ± 12 bpm (range, 44–90 bpm). The average denoising time for 0.9 mm^3^ isotropic resolution (Optimization 2 of PROST) was ∼25 s, whereas the average MR reconstruction time (Optimization 1 of PROST) was ∼1 min, resulting in a total average reconstruction time of ∼5 min 40 s for the whole 3D‐PROST reconstruction using 4 outer iterations.

### 3.1 Impact of undersampling on reconstruction

The average imaging time (min:s) for the fully sampled 3D CMRA acquisition with isotropic resolution 1.2 mm^3^ was 22:30 ± 4:54 with 100% scan efficiency, which was significantly reduced with 5‐fold undersampling (4:11 ± 1:03, *P* < 0.05) and 9‐fold undersampling (2:36 ± 0:24, *P* < 0.05).

**Figure 2:**
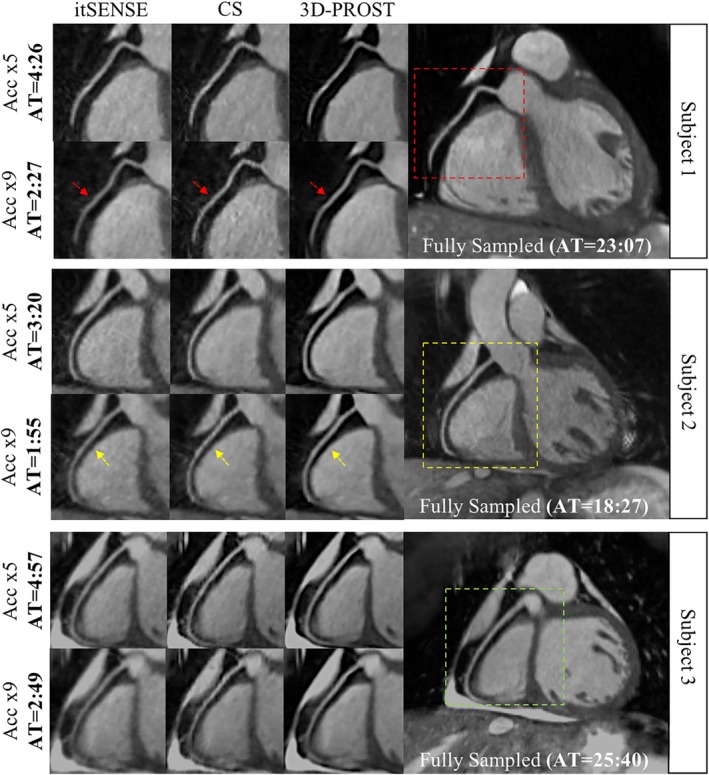
Example of reformatted images of the RCA from 3 representative healthy subjects acquired at 1.2 mm^3^ isotropic resolution with a fully sampled whole‐heart CMRA acquisition, and with 2 undersampled acquisitions (Acc × 5 and Acc × 9), reconstructed using iterative SENSE (itSENSE), wavelet‐based compressed‐sensing reconstruction (CS) and the proposed 3D patch‐based approach (3D‐PROST). All acquisitions were performed under free‐breathing with 100% respiratory efficiency. 3D‐PROST provides higher image quality and sharpness (red and yellow arrows) than itSENSE and CS for both acceleration factors, achieving similar image quality to the fully sampled reference. Acquisition times (AT) are expressed as min:s

Reformatted RCA images from 3 representative subjects are shown in Figure 2 for itSENSE, CS, and 3D‐PROST reconstructions in comparison to the reference fully sampled image. For both undersampling factors, CS and 3D‐PROST reduce blurring and suppress noise artifacts compared to itSENSE. 3D‐PROST further improved the delineation of the proximal segment of the RCA, achieving similar image quality to the fully sampled reference.

**Figure 3:**
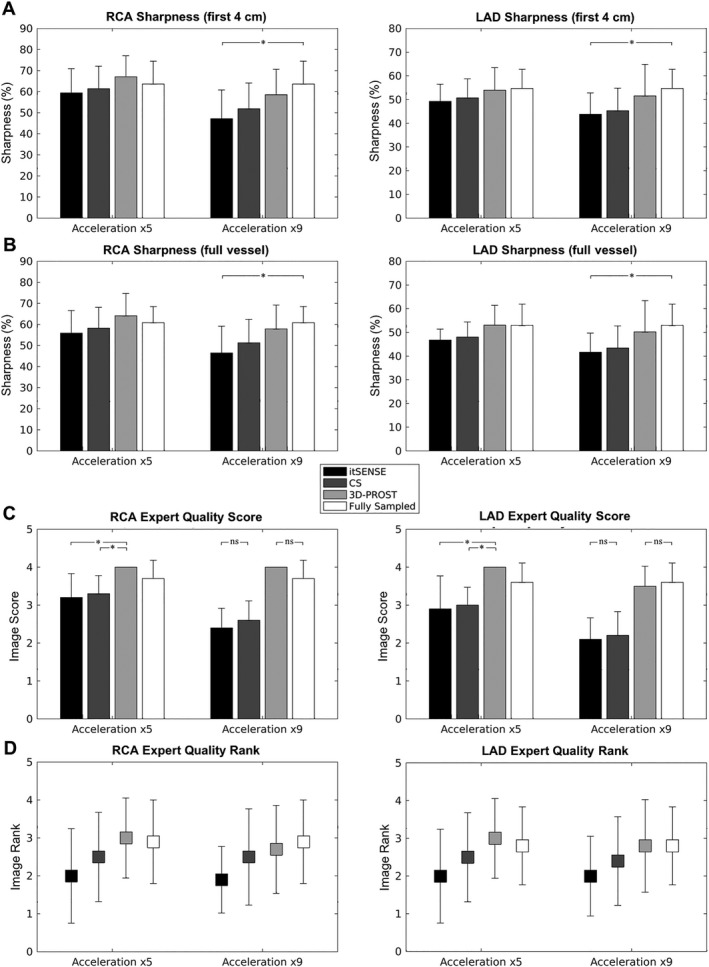
Quantitative coronary vessel sharpness for the first 4 cm (A) and the full length (B) and qualitative visual score (C) and image ranking (D) results from 10 healthy subjects who underwent 100% scan efficiency accelerated CMRA with isotropic resolution 1.2 mm^3^ and 2 different undersampling factors (×5 and ×9). A vessel sharpness of 100% marks an abrupt change in signal intensity whereas a sharpness of 0% indicates the absence of an edge. Results are expressed as mean ± SD. Differences with statistical significance are identified by *P < 0.016 (ns = not significant)

Vessel sharpness (first 4 cm and full length) of the LAD and RCA are summarized in Figure 3. Coronary vessel sharpness obtained using 3D‐PROST was higher compared with itSENSE and CS and as good, if not higher than the fully sampled reference for both the 5‐fold and 9‐fold undersampled scans (Figures 3A and 3B), in spite of a considerable reduction of the total scan times. The impact of 3D‐PROST on coronary vessel sharpness was particularly noted for high acceleration (×9) with sharpness in close agreement with the fully sampled reference, whereas itSENSE and CS failed to preserve the anatomical edges of the coronaries. The visual quality scores (Figure 3C) indicate that 3D‐PROST and fully sampled reconstructions have the best image quality, notably better than CS and itSENSE. Image rank of the RCA and LAD (Figure 3D) yielded similar values between 3D‐PROST and fully sampled reconstructions, differentiating them from CS and itSENSE for both accelerations.

### 3.2 Impact of resolution on coronary visualization

The average imaging time (min:s) for the 0.9 mm^3^ sub‐millimeter isotropic resolution 3D CMRA data was 7:57 ± 1:18 with 5‐fold undersampling and 4:35 ± 0:44 with 9‐fold undersampling with 100% scan efficiency.

**Figure 4:**
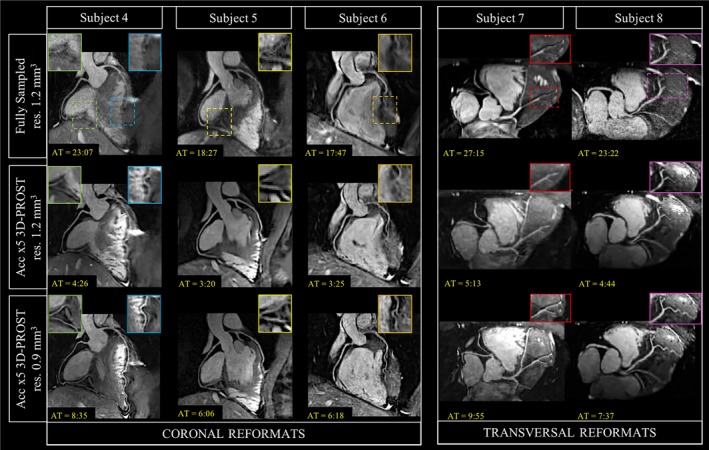
CMRA images of 5 representative healthy subjects reformatted along the LAD and RCA with fully sampled and 5‐fold undersampled 1.2 mm^3^ isotropic resolution and 5‐fold undersampled 0.9 mm^3^ isotropic resolution using the proposed acquisition and 3D‐PROST reconstruction approach. Close‐up views are shown for each reconstruction. Acquisition times (AT) are expressed as min:s

Representative CMRA reformats from 5 healthy subjects are shown in Figure 4 for fully sampled 1.2 mm^3^ resolution, × 5 undersampled 1.2 mm^3^ resolution and ×5 undersampled 0.9 mm^3^ resolution. The 5‐fold undersampled sub‐millimeter 3D‐PROST CMRA images provided the best image quality with clear delineation of the left coronary system and improved visualization of the distal segments. Note the presence of residual motion artifacts in the fully sampled experiments, particularly in subject 4, which may be associated with the long scan times. Vessel sharpness improvement at sub‐millimeter resolution is also observed. The distal portion of the LAD is better visualized with 0.9 mm^3^ isotropic resolution than with 1.2 mm^3^ isotropic resolution for both fully sampled and 5‐fold undersampled acquisitions, particularly in subjects 4 and 6. In subject 8, the sub‐millimeter resolution provides the best delineation of the first diagonal branch and its bifurcation of the LAD, although this segment appears blurred and hardly distinguishable in the lower resolution images.

**Figure 5:**
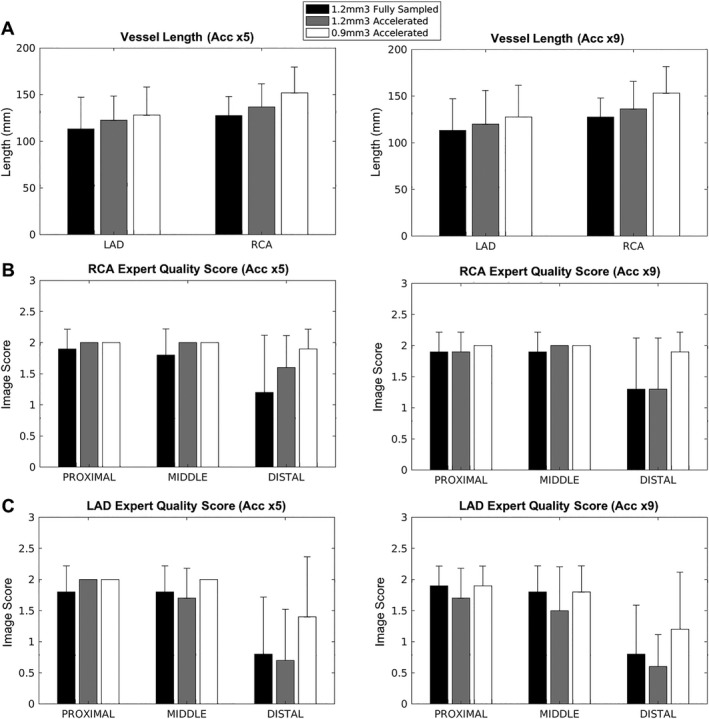
Quantitative coronary vessel length (A) and qualitative visual score of the RCA (B) and LAD (C) from ten healthy subjects (N=10) who underwent accelerated CMRA with isotropic resolution 1.2 mm^3^ and 0.9 mm^3^ and with two different undersampling factors (5 and 9). Results are expressed as mean ± standard deviation. Differences with statistical significance are identified by *P < 0.025 (ns = not significant)

The quantitative and qualitative evaluations for the fully sampled 1.2 mm^3^, × 5 undersampled 1.2 mm^3^ and ×5 undersampled 0.9 mm^3^ reconstructed images are provided in Figure 5. The visible lengths were higher with 0.9 mm^3^ isotropic resolution with both 5‐fold undersampling and 9‐fold undersampling than the fully sampled reference acquired at an isotropic resolution of 1.2 mm^3^, whereas the scan time was significantly shorter (5‐fold: 7:57 ± 1:18, 9‐fold: 4:35 ± 0:44 vs. fully sampled 22:30 ± 4:54). Vessel lengths obtained with 3D‐PROST at sub‐millimeter isotropic resolution and undersampling factor of 9 were higher than the lower‐resolution 1.2 mm^3^ isotropic resolution undersampled by a factor of 5 (Figure 5A), whereas the total scan times were similar (4:11 ± 1:3 vs. 4:35 ± 0:44). Coronary visualization of the proximal and middle segments of the RCA and LAD yielded similar values between the 1.2 mm^3^ and 0.9 mm^3^ isotropic acquisitions. However, the distal segments were better visualized with 0.9 mm^3^ isotropic resolution, even for highly accelerated acquisitions (Figures 5B and 5C).

**Figure 6:**
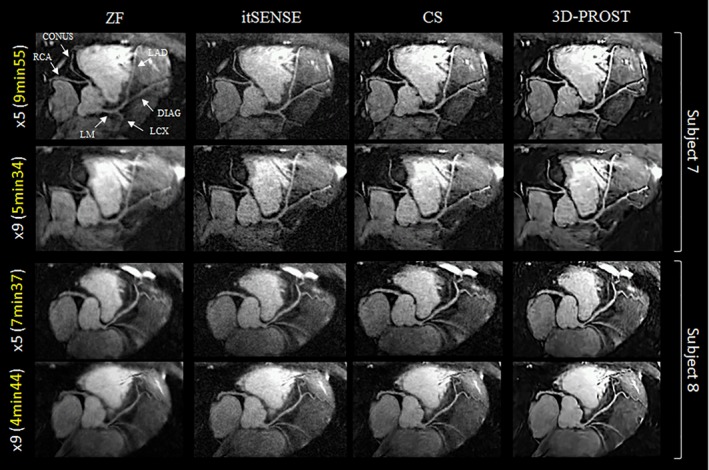
CMRA images reformatted along LAD for 2 representative healthy subjects. Acquisitions were performed with isotropic 0.9 mm^3^ resolution and 100% respiratory efficiency. Accelerated acquisitions with 2 different undersampling factors (×5 and ×9) are shown. Motion‐corrected images were reconstructed using zero‐filling (ZF), iterative SENSE (itSENSE), a Wavelet‐based compressed‐sensing reconstruction (CS), and the proposed 3D patch‐based technique (3D‐PROST). 3D‐PROST provides higher image quality than ZF, itSENSE and CS for both acceleration factors, with clear depiction of the left and right coronary systems as well as thin surrounding vessels (e.g., conus branching on Subject 7). Scan times are expressed as min:s. AT, acquisition time; RCA, right coronary artery; LAD, left anterior descending artery; LM, left main; LCX, left circumflex; DIAG, diagonal branch

Reformatted coronary artery images of 2 representative subjects acquired with sub‐millimeter resolution and 100% scan efficiency are shown in Figure 6 for zero‐filling (ZF), itSENSE, CS, and 3D‐PROST reconstructions. Visualization of the left coronary system was improved with itSENSE relative to the ZF reconstruction. Reconstruction with CS improved the visualization of the distal segments while reducing noise and preserving the underlying coronary structures. 3D‐PROST reconstruction led to substantial improvement in vessel sharpness and overall image quality. The accelerated sub‐millimeter images combined with 3D‐PROST reconstruction enable to capture the entire coronary tree and the surrounding vessels with high quality. Thin structures, such as the conus artery branching from the proximal RCA on subject 7 are intrinsically preserved, despite high acceleration factors (see Supporting Information Video S1).

## 4 DISCUSSION

In this study, we proposed a framework for highly accelerated sub‐millimeter free‐breathing 3D CMRA that combines 2D translational respiratory motion correction, with a variable density spiral‐like Cartesian trajectory and a novel 3D‐PROST reconstruction. The proposed approach enables sub‐millimeter CMRA acquisitions in a fast and predictable scan time. The use of iNAVs enables a substantial reduction of scan time compared to conventional dNAV scan, as previously shown,^31^, ^40^ whereas the use of the proposed acquisition trajectory and reconstruction enables further acceleration.

The performance and feasibility of the proposed framework were assessed in a phantom and in 10 healthy subjects. Phantom acquisitions were performed to evaluate the impact of undersampling on the reconstructed resolution. The undersampled variable density spiral‐like Cartesian sampling introduces incoherent noise‐like artifacts, which lead to robust patch matching during 3D‐PROST reconstruction and therefore high image quality reconstruction. At high undersampling, the fine structures of the resolution phantom were hardly detectable with zero‐filled reconstruction, but the use of similarity through 3D patch matching and low‐rank thresholding within 3D‐PROST led to successful 3D reconstructions with preservation of resolution as demonstrated by the measured MTF (see Supporting Information Figure S1).

The proposed 3D‐PROST reconstruction integrates self‐similarity information, by grouping 3D patches with similar structures. Low‐rank properties and sparsity of the group are enforced to reduce the noise of the reconstructed volume whereas the MR reconstruction step was used to recover an isotropic 3D volume and enforce data fidelity. An augmented Lagrangian formulation was used to efficiently decompose the main cost function into 2 sub‐problems that have straightforward solutions. The improved performance can be explained by the fact that CMRA images contain a rich amount of correlated 3D structures and therefore high sparsity degree can be achieved by merging this information. The increased sparsity of the proposed framework promotes superior denoising and structures recovery in comparison to other established approaches such as itSENSE or CS. Similar image quality was achieved with the proposed framework in comparison to the reference fully sampled acquisition for an isotropic 1.2 mm^3^ resolution.

The significant scan time reduction achieved with the proposed framework was exploited to enable sub‐millimeter (0.9 mm^3^) whole‐heart Cartesian CMRA in clinically feasible scan times. Although comparable visualization of the proximal and middle segments was observed between 1.2 mm^3^ and 0.9 mm^3^ isotropic resolutions, visualization of the distal coronary segments was further improved at 0.9 mm^3^ isotropic resolution, even for highly accelerated acquisitions. In some cases, the proposed approach outperformed the image quality of the fully sampled 1.2 mm^3^ acquisition in terms of sharpness. This may be partly explained by the significantly longer scan times of the fully sampled acquisitions that are more prone to motion artifacts because of considerable drift in breathing pattern during imaging as well as non‐rigid cardiac deformations that propagate over the acquisition.^41^, ^42^ Conversely, the proposed approach enables the acquisition of 3D CMRA data in a relatively short time period, therefore reducing the susceptibility to cardiac motion and respiratory drift.

A limitation of the present study is that, although 2D image‐based navigation enables 100% scan efficiency and drastically reduces the scan time, this technique only enables 2D translational respiratory motion correction. However, this issue was not shown to be significant in the present work when acquiring sub‐millimeter resolution data set in healthy subjects. This limitation can be overcome by incorporating non‐rigid motion correction of respiratory motion in the reconstruction problem, as reported in previous studies.^31^, ^40^, ^43^ The design of 3D‐PROST as a separate 3D patch‐based optimization and a MR reconstruction problem enables the straightforward integration of non‐rigid motion in the acquisition operator *E*, without affecting the 3D patch‐based denoising step. This formulation would greatly benefit from an increase in image quality, particularly in patients with irregular breathing patterns, at the expense of increased reconstruction times. The impact of integrating non‐rigid motion‐correction into accelerated sub‐millimeter isotropic CMRA acquisitions has not yet been established and is currently under investigation. Furthermore, arrhythmia detection/rejection approaches^44^, ^45^ can be included to further improve image quality and prevent residual cardiac motion artefacts because of irregular R–R intervals. Additionally, 3D image‐based navigators can be implemented and used to estimate beat‐to‐beat 3D translational displacements and to correct k‐space data for respiratory motion,^13^, ^14^ however, they may lead to longer delays between preparation pulses and the 3D CMRA acquisition.

The proposed reconstruction method iterates between self‐similarity extraction, rank reduction, and MR reconstruction. Although motion correction was performed in‐line with the scanner software, integration of the proposed 3D‐PROST technique for in‐line reconstruction is currently under development. Reconstruction times were in the order of 5 min for each data set. The formulation of the 3D‐patch extraction and thresholding as reported in this article strongly enables the use of graphic processing unit or clusters, which should therefore offer the advantage of sub‐minute 3D reconstructions. Future work will investigate these implementations to facilitate clinical translation.

Our technique could have direct clinical implications as it offers several potential advantages: (1) high image quality and high‐resolution CMRA reconstruction by exploiting the inherent 3D redundancy of the coronary anatomy structure, (2) high acceleration can be achieved leading to short scan times, improved patient comfort and reduction of respiratory motion artifacts, and (3) fast and efficient implementation leading to clinically feasible reconstruction times. Although the results reported here are encouraging, further studies in larger patient cohorts are needed. Substantial clinical evaluations will help to verify the efficiency of the proposed framework. We also anticipate that this technique will enable the acceleration of other recently developed CMRA sequences. For example, techniques that simultaneously provide bright‐blood and black‐blood whole‐heart data 46 or black‐blood LGE and bright‐blood CMRA^47^ can be markedly accelerated and could be integrated into clinical routine using the present framework.

The performance of the proposed framework suggests an opportunity to reach even higher resolutions in the future, which will be crucial to accurately detect and characterize luminal stenosis in patients with coronary artery disease. For example, at 0.7 mm^3^ isotropic resolution, a 9‐fold accelerated 3D CMRA scan using the proposed framework should take ∼9 min (correspondingly, ∼1.3 h for a fully sampled acquisition with 100% scan efficiency). Future work will investigate this extension.

## 5 CONCLUSION

We demonstrate the feasibility of combining an efficient variable density Cartesian sampling trajectory with 2D iNAV‐based translational motion correction and 3D‐PROST undersampled reconstruction to obtain isotropic sub‐millimeter 3D coronary images under free‐breathing in ∼5 min predictable scan time, which is crucial for its integration in routine clinical examinations. Ultimately, this technique might be useful for rapid screening of the major coronary vessels in patients with suspected coronary artery disease. Further clinical validation is now warranted.

## CONFLICT OF INTEREST

Dr. Neji Radhouene is employed by Siemens Healthcare.

## Supporting information


**FIGURE S1** Examples for the application of the proposed highly undersampled 3D‐PROST approach on a high‐resolution phantom. Fully sampled acquisition (FS‐left column) is compared to 5‐fold and 9‐fold accelerated acquisitions (middle and right columns respectively), for both zero‐filled (ZF‐top row) and 3D‐PROST (middle row) reconstructions. The variable density undersampled Cartesian images with ZF reconstruction show significant blurring and contrast loss, while the 3D‐PROST images exhibit sharp edges with faithful preservation of small details (as shown on the cross‐section profiles). Reconstructed resolution for ZF and the proposed 3D‐PROST technique are shown on the top left corner (modulation transfer function (MTF) profiles are taken in the pink box). Differences with statistical significance are identified by *P < 0.05 (vs. FS)

**FIGURE S2** Coronal (A), sagittal (B), and transversal (C) views of CMRA images reconstructed using iterative SENSE (itSENSE), the proposed framework with 2D‐patches of size 5 × 5 voxels and a search window of 14 × 14 voxels (2D‐PROST) and 3D‐patches of size 5 × 5 × 5 voxels with a search window of 14 × 14 × 14 voxels (3D‐PROST). The 3D CMRA acquisition was performed in free‐breathing in 1 healthy subject with an undersampling factor of 5% and 100% scan efficiency. Images reformatted along the left (LAD) and right (RCA) coronary arteries are shown on the right. 3D‐PROST provides better image quality than 2D‐PROST, reducing streaking artifacts (yellow arrows). RV, right ventricle; RCA, right coronary artery; LAD, left anterior descending artery; PA, pulmonary artery; SVD, superior vena cava; AO, aorta; LM, left main; LCX, left circumflex; PT, pulmonary trunk
**VIDEO S1** Example reconstructions from 2 healthy subjects with acceleration ×9 (total acquisition times of 3:59 and 4:30 [min:s]) and isotropic resolution 0.9 mm^3^ are shown. Increasing image resolution to sub‐millimeter isotropic voxels with the proposed variable density Cartesian sampling and 3D‐PROST reconstruction allows reliable depiction of extensive portions of the right and left coronary systems
