# Supplementary material for: Five‐minute whole‐heart coronary MRA with sub‐millimeter isotropic resolution, 100% respiratory scan efficiency, and 3D‐PROST reconstruction
Source: Magn Reson Med. 2018 Jul 29;81(1):102–15. doi: 10.1002/mrm.27354 (PMC6617822; doi:10.1002/mrm.27354)
Supplement: Supplementary file 2 — FIGURE S2 Coronal (A), sagittal (B), and transversal (C) views of CMRA images reconstructed using iterative SENSE (itSENSE), the proposed framework with 2D‐patches of size 5 × 5 voxels and a search window of 14 × 14 voxels (2D‐PROST) and 3D‐patches of size 5 × 5 × 5 voxels with a search window of 14 × 14 × 14 voxels (3D‐PROST). The 3D CMRA acquisition was performed in free‐breathing in 1 healthy subject with an undersampling factor of 5% and 100% scan efficiency. Images reformatted along the left (LAD) and right (RCA) coronary arteries are shown on the right. 3D‐PROST provides better image quality than 2D‐PROST, reducing streaking artifacts (yellow arrows). RV, right ventricle; RCA, right coronary artery; LAD, left anterior descending artery; PA, pulmonary artery; SVD, superior vena cava; AO, aorta; LM, left main; LCX, left circumflex; PT, pulmonary trunk VIDEO S1 Example reconstructions from 2 healthy subjects with acceleration ×9 (total acquisition times of 3:59 and 4:30 [min:s]) and isotropic resolution 0.9 mm3 are shown. Increasing image resolution to sub‐millimeter isotropic voxels with the proposed variable density Cartesian sampling and 3D‐PROST reconstruction allows reliable depiction of extensive portions of the right and left coronary systems [file MRM-81-102-s002.docx]

**Supporting Material 1**

***Phantom study***

*Acquisition:* A phantom acquisition was performed to evaluate the impact of highly undersampled acquisitions on the reconstructed resolution. The phantom was composed of parallel bar patterns with in-plane resolution of 1 mm. The relevant imaging parameters were as follows: 3D bSSFP sequence, field-of-view (FOV) = 306 x 306 x 80 mm^3^, TE = 1.63 ms, TR = 3.7 ms, flip angle (FA) = 90°, 0.9 mm^3^ isotropic resolution, receiver bandwidth = 875 Hz/pixel, T2-preparation duration = 40 ms. Acquisitions were performed with undersampling factors of 5 and 9. Additionally, a fully-sampled acquisition was performed with the same resolution for comparison purposes.

*Data Analysis*: The modulation transfer function (MTF) was used to quantify the spatial resolution for the undersampled and fully-sampled reconstructions (48). The MTF was directly obtained using a slanted edge method. A profile (edge spread function) was taken along a sharp edge of the phantom, then differentiated to obtain the associated line spread function, and finally fast Fourier transformed to get the final MTF, which was then normalized. The reconstructed resolution was given by the value of the MTF at 10%. The MTF was obtained from 20 intensity profiles taken on the reference fully-sampled image, the zero-filled (ZF) reconstruction and the 3D-PROST reconstructions. The average MTFs were compared for both accelerations. A paired two-tailed Student t-test was used for statistical analysis, with *P* < 0.05 considered statistically significant.

***Results***

The total scan times (min:sec) were 11:30 (fully-sampled), 2:13 (5-fold acceleration) and 1:15 (9-fold acceleration) with isotropic resolution of 0.9 mm^3^.

The reconstructed images using ZF and 3D-PROST are shown in Fig. S1 in comparison with the reference fully-sampled image. The measured MTFs are shown on the top of each image. Significant aliasing artifacts can be observed on the ZF images, and were particularly pronounced for high undersampling where the depiction of the bar pattern becomes indistinguishable. Conversely, high image quality can be appreciated using the proposed 3D-PROST reconstruction, with clear and sharp depiction of the phantom structures with high spatial resolution, even for high acceleration (x9), providing excellent visual agreement with the reference fully-sampled image. This observation is confirmed and illustrated in Fig. S1 (bottom) where the intensity profiles taken perpendicularly across the pattern are plotted for each reconstruction. The fine and sharp structures of the bars are well preserved with 3D-PROST reconstruction while ZF reconstruction exhibits blurring and loss of contrast. The spatial resolution, as quantified by the MTF, was preserved with the proposed approach, showing no statistical differences with the reference fully-sampled image (5-fold: 0.90 ± 0.01, *P* = 0.31 and 9-fold: 0.90 ± 0.02, *P* = 0.17).


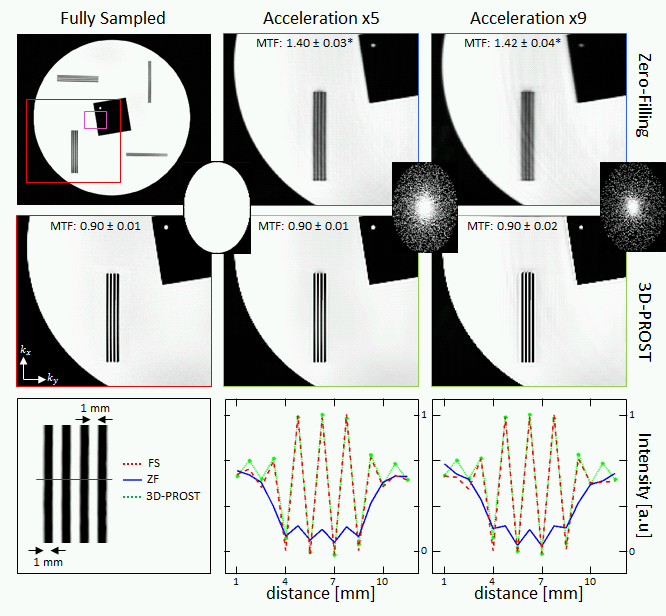


**Figure S1:** Examples for the application of the proposed highly undersampled 3D-PROST approach on a high-resolution phantom. Fully-sampled acquisition (FS-left column) is compared to 5-fold and 9-fold accelerated acquisitions (middle and right columns respectively), for both zero-filled (ZF-top row) and 3D-PROST (middle row) reconstructions. The variable density undersampled Cartesian images with ZF reconstruction show significant blurring and contrast loss, while the 3D-PROST images exhibit sharp edges with faithful preservation of small details (as shown on the cross-section profiles). Reconstructed resolution for ZF and the proposed 3D-PROST technique are shown on the top-left corner (modulation transfer function (MTF) profiles are taken in the pink box). Differences with statistical significance are identified by *P < 0.05 (versus FS).

**Supporting Material 2**

***2D-PROST vs. 3D-PROST***

In a single subject, 0.9 mm^3^ isotropic resolution undersampled data was also reconstructed with 2D patch-based low-rank reconstruction (2D-PROST) to investigate the effect of exploiting 3D redundancy. 2D-PROST reconstruction was performed with the same parameters as 3D-PROST except for the size of the patches $n$ (2D: 5x5 voxels vs. 3D: 5x5x5 voxels) and the selection window $d$ (2D: 14x14 voxels vs. 3D: 14x14x14 voxels). The 2D patches were taken along the coronal direction.

***Results***

Figure S2 shows the reconstruction results of a whole-heart 3D CMRA scan accelerated with an undersampling factor of 5 at 0.9 mm^3^ isotropic resolution. Example slices of CMRA images and the reformatted coronal and sagittal planes are shown for itSENSE, 2D-PROST and the proposed 3D-PROST reconstructions. Both images reconstructed with PROST show improved visual image quality compared to itSENSE. However, residual streaking artifacts can be seen on the 2D-PROST images, particularly in the sagittal and transversal slices, thus affecting the ability to accurately track the mid-segment of the RCA (yellow close-up views). These artefacts are particularly visible on the reformatted CMRA images, degrading the visibility of the LAD and exhibiting artefacts which could be misinterpreted as stenosis (yellow arrows). Most artefacts are removed by exploiting the 3D nature of the CMRA images with the proposed 3D-PROST reconstruction, achieving better image quality and clear delineation of the coronary segments.


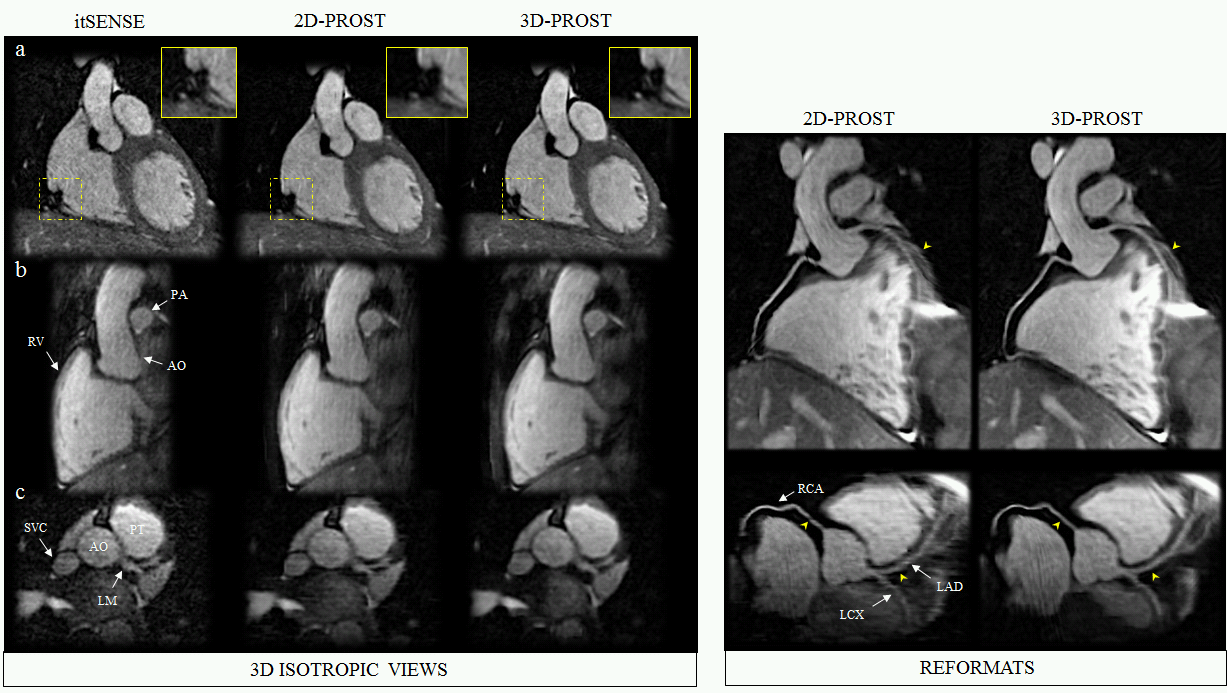


**Figure S2:** Coronal (a), sagittal (b) and transversal (c) views of CMRA images reconstructed using iterative SENSE (itSENSE), the proposed framework with 2D-patches of size 5x5 voxels and a search window of 14x14 voxels (2D-PROST) and 3D-patches of size 5x5x5 voxels with a search window of 14x14x14 voxels (3D-PROST). The 3D CMRA acquisition was performed in free-breathing in one healthy subject with an undersampling factor of 5 and 100% scan efficiency. Images reformatted along the left (LAD) and right (RCA) coronary arteries are shown on the right. 3D-PROST provides better image quality than 2D-PROST, reducing streaking artifacts (yellow arrows). RV = right ventricle, RCA = right coronary artery, LAD = left anterior descending artery, PA = pulmonary artery, SVD = superior vena cava, AO = aorta, LM = left main, LCX = left circumflex, PT = pulmonary trunk.
